# Value of Peer Mentoring in Advancing Science Careers: Lessons Learned From NR Impact

**DOI:** 10.1210/jendso/bvaf005

**Published:** 2025-01-13

**Authors:** Zeynep Madak-Erdogan, Rebecca B Riggins, Matthew J Sikora

**Affiliations:** Department of Food Science and Human Nutrition, University of Illinois at Urbana-Champaign, Urbana, IL 61801, USA; Department of Oncology, Lombardi Comprehensive Cancer Center, Georgetown University, Washington, DC 20057, USA; Department of Pathology, University of Colorado Anschutz Medical Campus, Aurora, CO 80045, USA

**Keywords:** nuclear receptors, near/peer-mentoring, early career and mid-career investigators

Principal investigators (PIs) who run an active research laboratory, teach regularly, and manage service commitments have always benefited from the support of a brain to pick, an ear to listen, and a push in the right direction. Finding this support is often challenging, but imagine if such support could come from a collective of ∼40 other PIs who have a deep understanding of the challenges early and mid-career scientists face within the same scientific field.

We underestimated the power of such a collective peer-mentoring group when we officially established NR IMPACT in 2019 [1]. Our aim was to start a collaborative networking group for early and mid-career scientists who study nuclear receptors. We aimed to foster purposeful and meaningful connections among NR IMPACT members that would translate into productive collaborations, joint publications, and mutual professional growth. However, in addition to these expected outcomes, NR IMPACT has also emerged as a valuable source of psychosocial support and belonging, which has proven to be indispensable in sustaining our career trajectories, particularly against the backdrop of the COVID-19 pandemic.

It quickly became clear that in addition to a passion for science, individuals in this group care about each other's professional development. The environment of trust, respect, scientific synergy, and collective experience in various aspects of running a lab offered by NR IMPACT has been the difference between being well or unwell during challenging times. The close-knit nature of the group has also been instrumental in sharing the tacit knowledge of how to manage it all between more experienced and novice members of the group. Every member of NR IMPACT has something important to offer to the group. Because of the invaluable support that members receive, our members have a mutual growth and “buy-in” mindset and volunteer their time to contribute to the group to the best of their expertise. This strong support network leverages the greater availability of peers and near-peers, compared to senior mentors. As such, our group also covers a much wider variety of issues needed at different stages of academic careers and provides much-needed constructive feedback on new project ideas, manuscripts, and grant applications. This safety net built on mutual respect and trust helps us collectively reflect on decisions, as group members share their strategies or challenges that they encountered when handling similar situations.

In 2022, we held our first in-person meeting at the Huntsman Cancer Institute, where we had 3 full, wonderful days dedicated to nuclear receptor science and collaboration. We recently convened for our second in-person meeting at the University of Illinois Urbana-Champaign, further pollinating ideas across nuclear receptor science and engineering. In the meantime, we continue to meet biweekly virtually to present scientific talks, discuss policy issues, and share our challenges with (and solutions to) the current system. As the collective expertise in our group diversifies, our shared goals and experiences help our members to consider bigger scientific projects. The success of our group is also evidenced by our outcomes. Our group has been awarded 5 collaborative co-PI grants and published 15 peer-reviewed publications. Our networking has led to >20 invited seminars at member institutions and extensive leadership opportunities at research conferences chaired or organized by members. However, we continuously evaluate how NR IMPACT can better serve members and our nuclear receptor research programs. In the past 2 years, NR IMPACT has developed broader scientific expertise in the group through targeted recruitment, established a shared values document, and formed working groups for new initiatives, all to support long-term growth. Most recently, we reasoned that NR IMPACT is well-placed to engage postdoctoral fellows on the faculty job market, providing them with vital support at a very critical point in their careers. This, in turn, has inspired conversations about when and how to establish an “emeritus” role for NR IMPACT members whose trainees become faculty themselves, such that the newest members of this community can forge their own path while benefitting from the same supportive community that sustained us. We aspire to build a pipeline that fosters the continued growth of nuclear receptor research as a robust community.

Gorelick et al [2] reviewed the progress of NR IMPACT and discussed how this peer-mentoring cohort is removing hurdles for new faculty and advancing nuclear receptor biology in detail. We posit that a similar model for small collective peer mentoring groups can be used by scientists in other research disciplines, yet there are challenges in group development. Importantly, we found that despite technology bringing us together, time zones place real constraints on “real-time” member engagement at scheduled events. Similarly, we all face distinct challenges in navigating local, regional, or international funding and career development systems, and peer mentoring groups should have critical mass to address those specific contexts. Critical mass comes at a cost to managing group size—also a major consideration, since trust and a level of closeness between members is a paramount feature of peer mentoring. Despite these challenges, small-scale peer-mentoring models offer a unique safety net and provide the advantage of being supported by colleagues across shared career trajectories. We continue to learn and reap the benefits of being in such a group every day and hope that the model developed by NR IMPACT proliferates across other disciplines.

## Disclosures

Z.M.E. is editor-in-chief and R.B.R. is an associate editor of the *Journal of the Endocrine Society*. The other authors have no other relevant disclosures.
